# A Giant, Neglected Leiomyosarcoma on the Left Shoulder

**DOI:** 10.7759/cureus.59081

**Published:** 2024-04-26

**Authors:** Sehrish Noorali, Damian Casadesus, Sandra Kaldas, Mingyi Zhang

**Affiliations:** 1 Internal Medicine, Ross University School of Medicine, Miami, USA; 2 Internal Medicine, Jackson Memorial Hospital, Miami, USA; 3 Surgery, Nanjing Medical University, Nanjing, CHN

**Keywords:** dystonia upper extremity, upper extremity sarcoma, upper limb surgery, neglect, leiomyosarcoma of somatic soft tissues

## Abstract

This paper examines the impact of delayed diagnosis and treatment on the prognosis of patients with leiomyosarcomas (LMS). We present a case study highlighting the consequences of neglected LMS, focusing on vascular involvement and metastatic potential. Our findings underscore the importance of early detection and intervention in improving patient outcomes.

Additionally, we discuss the challenges associated with diagnosing rare skin LMS and the implications of limited access to medical screening. Through a comprehensive analysis of the literature, we elucidate the critical role of routine surveillance in detecting these malignancies at an earlier stage, thus facilitating timely intervention and potentially curative treatment. This study underscores the urgency of raising awareness among both healthcare providers and the general population about the significance of early detection and prompt management in mitigating the adverse outcomes associated with neglected LMS.

## Introduction

Leiomyosarcomas (LMS) represent a rare subset of malignant neoplasms originating from smooth muscle cells, boasting a diverse anatomical distribution spanning various bodily compartments. Predominantly observed in the uterus, gastrointestinal tract, and retroperitoneum, the estimated annual incidence of LMS stands at a modest one to two cases per 100,000 individuals, with a noted propensity toward afflicting women during the perimenopausal phase [1]. Notably, the morphological spectrum of LMS in women tends to encompass retroperitoneal and vascular-associated variants, compared with a predominance of non-cutaneous soft tissue and cutaneous manifestations in their male counterparts. The etiological underpinnings of LMS remain elusive, albeit with discernible associations with specific genetic predispositions such as hereditary retinoblastoma, Li-Fraumeni syndrome, and neurofibromatosis type 1, alongside environmental determinants like prior radiation exposure and select chemical insults.

Symptomatology in LMS is contingent upon tumor size and anatomical localization, with clinical presentations ranging from constitutional manifestations, such as weight loss, fatigue, and fever, to gastrointestinal disturbances like nausea and vomiting. Initial stages of LMS often unfold surreptitiously, manifesting as asymptomatic entities, only to escalate in severity as tumorigenesis progresses, potentially culminating in hepatic or pulmonary metastases. Prompt surgical excision remains the cornerstone of therapeutic intervention, aimed at averting the apparition of disease recurrence.

Diagnosis of LMS hinges upon a multimodal approach, incorporating high-resolution imaging modalities, including computed tomography (CT), magnetic resonance imaging (MRI), and ultrasonography. While MRI boasts superior delineation of extremity and head-neck lesions, CT excels in characterizing retroperitoneal and visceral counterparts. Definitive confirmation necessitates histopathological scrutiny via biopsy, facilitating precise identification of the neoplastic identity.

## Case presentation

A 55-year-old gentleman, a former captain of a ship in the Bahamas, burdened by a medical history encompassing coronary artery disease, hyperlipidemia, and iron deficiency anemia, sought refuge within our institution's emergency department. His narrative unveiled a seemingly innocuous dermal anomaly, initially perceived as a mole four years prior, turned into a formidable mass on the top of his left shoulder, attaining proportions to a "cantaloupe" and accompanied by an unsettling mucopurulent discharge. This mass inflicted a profound toll upon his daily endeavors, precipitating a noteworthy weight loss exceeding 40 pounds, as per the patient's history. Moreover, the history of familial malignancies, including breast and lung cancer within his maternal lineage, prostate cancer afflicting his sibling, and colorectal cancer in his paternal lineage, cast a shadow over his medical history.

**Figure 1:**
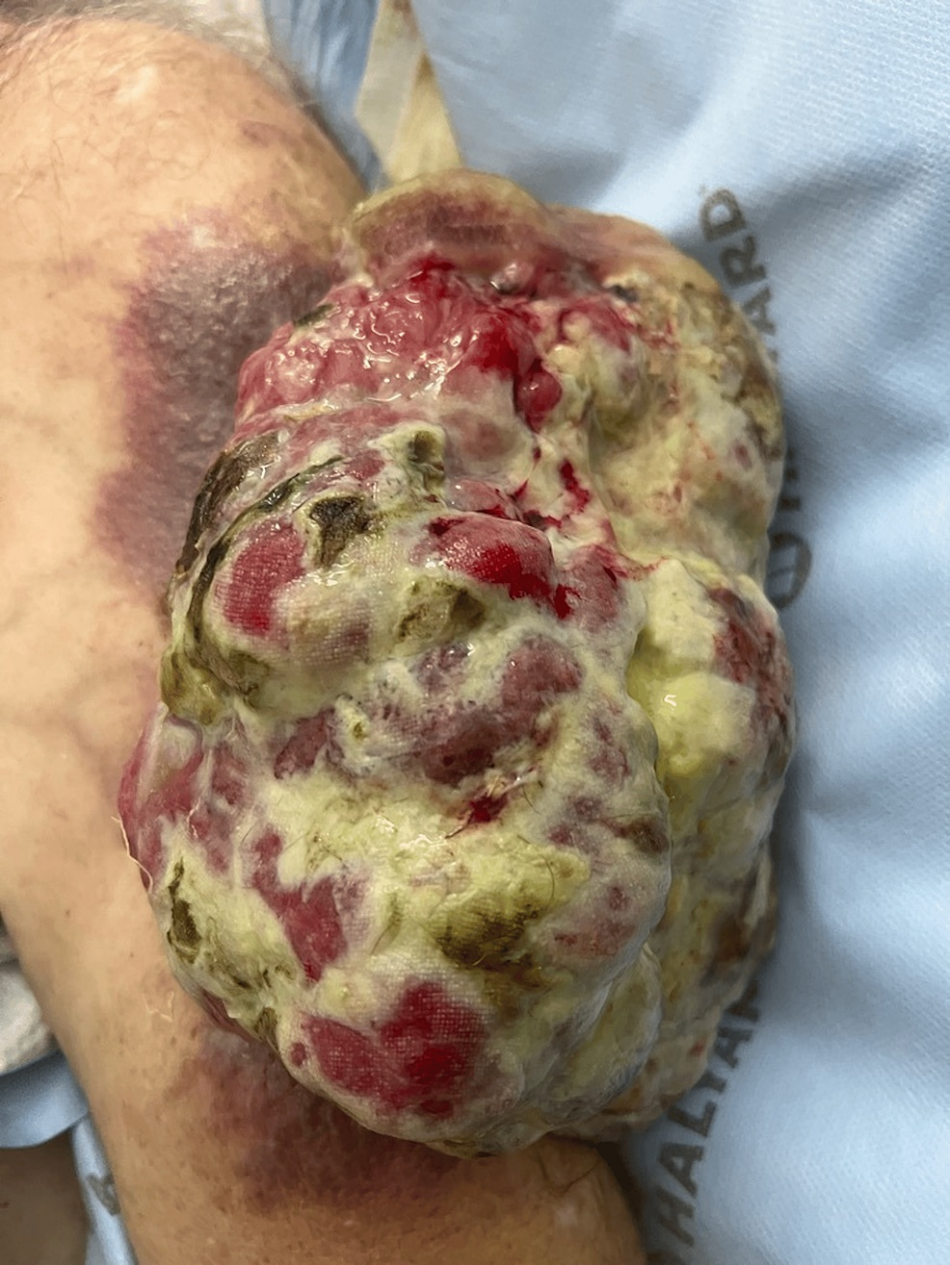
Leiomyosarcoma spanning approximately 15 x 15 cm.

Having traversed geographical bounds to another medical facility, he found himself under our care subsequent to an encounter revealing anemia. At our institution, his vital signs were stable upon presentation. A meticulous physical examination of the cardiopulmonary system and abdomen was normal, while an inspection of the patient's left upper arm unveiled a fungating mass, spanning approximately 15 x 15 cm, draining a mucopurulent discharge, as depicted in Figure 1.

**Figure 2:**
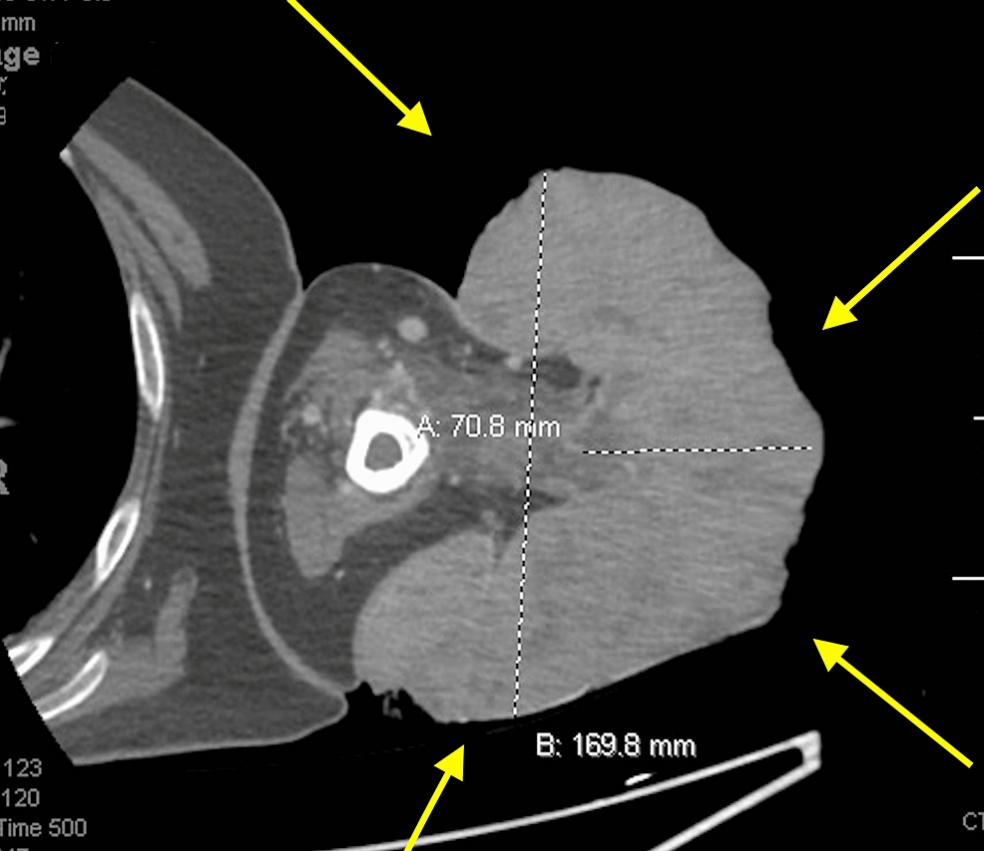
Upper extremity CT with contrast, unveiling a substantial pedunculated fungating mass, measuring approximately 17 x 14 x 8 cm, originating from the proximal lateral arm.

Upon initial assessment, the patient underwent upper extremity CT with contrast, unveiling a substantial pedunculated fungating mass measuring approximately 17 x 14 x 8 cm and originating from the proximal lateral arm (Figure 2). Notable areas of hypo-enhancement, indicative of internal necrosis and cystic alterations, dotted the mass. Moreover, vascularization of the lesion was pronounced, with multiple feeder arteries stemming from the brachial artery, accompanied by a network of recruiting veins from the cephalic vein.

Subsequent deep tissue biopsy yielded a positive diagnosis of LMS, characterized by immunopositivity for desmin, p63, and CD10 markers. Further imaging via upper extremity MRI without contrast delineated a conspicuous exophytic fungating mass, measuring 21.7 x 15.6 x 13 cm, with evident tethering of deltoid muscle fibers to the deep surface of the pedunculated tumor via a soft tissue stalk.

Concurrent evaluation via chest CT unveiled left axillary lymph nodes, measuring up to 1.6 cm in diameter. Subsequent biopsy of the left axillary lymph nodes revealed no discernible evidence of metastatic spread or malignancy. Confirmation of tumor dimensions via chest CT underscored a substantial mass measuring 24.5 x 15.8 x 15 cm.

**Figure 3:**
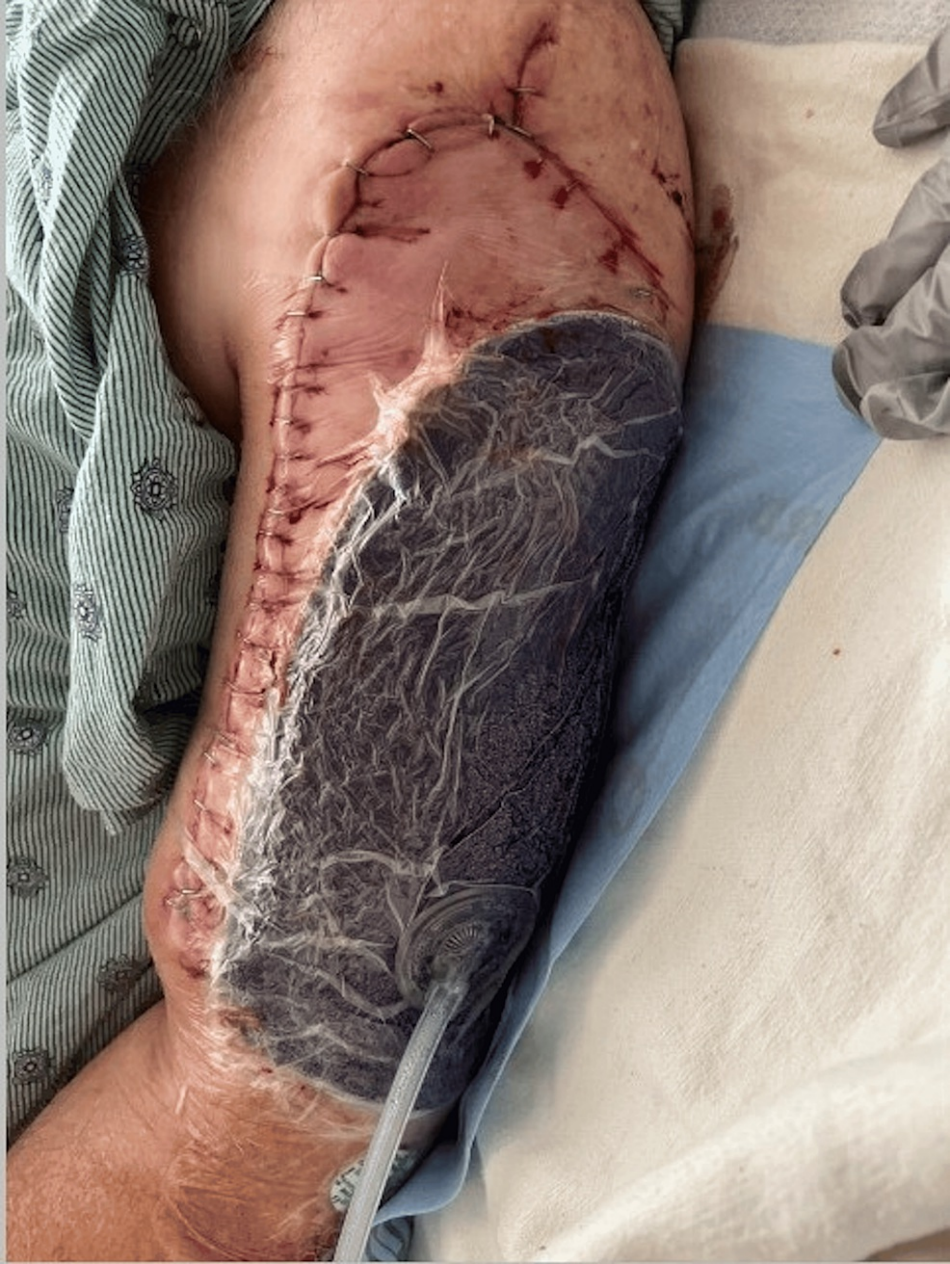
Wide local excision of the fungating soft tissue with left arm reconstruction with a latissimus dorsi myocutaneous flap into split-thickness skin graft measuring 8 x 12 cm.

The clinical discussion was mainly on treatment regimens such as amputation of the arm or tumor resection, followed by chemotherapy as part of his long-term care. The patient underwent wide local excision of the fungating soft tissue with left arm reconstruction with a latissimus dorsi myocutaneous flap into a split-thickness skin graft measuring 8 x 12 cm (Figure 3). The tumor resection biopsy confirmed the diagnosis of LMS. The utilization of negative pressure wound therapy reduces the risk of postoperative wound complications in patients with surgical tumor resections of soft tissue sarcomas. The patient recovered well from surgery and was discharged with follow-up, specifically for subsequent chemotherapy.

## Discussion

The clinical manifestation of LMS varies depending on the tumor's site and dimensions. In the uterus, symptoms may encompass abnormal vaginal bleeding, pelvic discomfort, and the presence of a palpable mass. Gastrointestinal involvement can manifest as abdominal pain, gastrointestinal bleeding, and bowel obstruction. Retroperitoneal LMS often elicits back pain and the discovery of an abdominal mass. Conversely, LMS in the extremities may induce pain, edema, or neurological deficits contingent upon the lesion's size and depth.

Upon assessment, a staggering 90% of patients receive a diagnosis of grade 2 or 3 cancer. Notably, tumor location emerges as an autonomous prognostic determinant, with upper extremity-based LMS demonstrating a comparatively favorable outcome juxtaposed with retroperitoneal counterparts. However, this assertion is underpinned by limited study cohorts, necessitating cautious interpretation. LMS originating from vascular surfaces typically portend a dire prognosis, rendering them surgically unresectable [1]. Depth of tumor infiltration poses another significant prognostic variable, with deeper lesions often yielding poorer outcomes due to surgical complexities and potential vascular compromise [2]. Complications of LMS hinge on its anatomical site, potentially precipitating mass effects via external compression and facilitating early metastatic dissemination [3].

Therapeutic interventions typically entail surgical excision of the tumor, complemented by adjuvant radiation or chemotherapy, contingent upon disease extent, patient health status, and tumor characteristics. Recent meta-analytical findings advocate for a therapeutic regimen comprising doxorubicin and ifosfamide as frontline agents, followed by monotherapy with ifosfamide, anthracycline, or trabectedin, which has demonstrated superior overall and progression-free survival rates [1]. Additionally, studies underscore the adverse impact of tumor size exceeding 5 cm and positive resection margins on overall survival, while advocating for adjuvant radiotherapy to mitigate metastatic spread [4].

Prognostically, LMS portends a bleak outlook, with a five-year survival rate hovering around 50% [5]. Prognostic determinants encompass tumor attributes, disease extent, and patient well-being. However, recent investigations suggest a relatively low incidence of local recurrence (12-15%) coupled with a modest rate of distant metastasis (29-35%), offering a glimmer of hope amidst the prevailing gloom [6].

## Conclusions

The delayed diagnosis and medical treatment in our patient luckily did not have a detrimental effect on his survival. Despite extensive vascular involvement from the brachial artery and cephalic vein, our patient, who has had extremity LMS for over four years, showed no signs of metastatic lesions. While enlarged lymph nodes were detected in the left axillary area, a biopsy revealed no malignancy. Early detection of the tumor could have improved the prognosis, but unfortunately, the patient did not seek medical attention. Patients with LMS are at high risk of recurrence due to vascular involvement of the tumor. Despite extensive vascular involvement in this case, our patient was fortunate to have no metastatic disease even after four years. He will continue to follow up with his doctors and finish rehabilitation before returning to his family in the Bahamas.
